# Modeling trauma in rats: similarities to humans and potential pitfalls to consider

**DOI:** 10.1186/s12967-019-2052-7

**Published:** 2019-09-05

**Authors:** Birte Weber, Ina Lackner, Melanie Haffner-Luntzer, Annette Palmer, Jochen Pressmar, Karin Scharffetter-Kochanek, Bernd Knöll, Hubert Schrezenemeier, Borna Relja, Miriam Kalbitz

**Affiliations:** 1 Department of Traumatology, Hand-, Plastic-, and Reconstructive Surgery, Center of Surgery, University of Ulm Medical School, Albert-Einstein-Allee 23, 89081 Ulm, Germany; 2 Institute of Orthopedic Research and Biomechanics, University Medical Center Ulm, Ulm, Germany; 3 Institute of Clinical and Experimental Trauma-Immunology, University of Ulm, Ulm, Germany; 4 Institute of Dermatology and Allergology, University Medical Center, University of Ulm, Ulm, Germany; 5 Institute of Physiological Chemistry, University of Ulm, Ulm, Germany; 6 Institute of Transfusion Medicine, University of Ulm and Institute of Clinical Transfusion Medicine and Immunogenetics Ulm, German Red Cross Blood Transfusion Service Baden-Württemberg – Hessen and University Hospital Ulm, Ulm, Germany; 7 Department of Trauma, Hand and Reconstructive Surgery, Goethe University Frankfurt, Frankfurt, Germany; 8 Department of Radiology and Nuclear Medicine, Experimental Radiology, Otto-von-Guericke University, Magdeburg, Germany

**Keywords:** Trauma research, Comparability, Translational research, Rat model, Translation pitfalls, Polytrauma

## Abstract

Trauma is the leading cause of mortality in humans below the age of 40. Patients injured by accidents frequently suffer severe multiple trauma, which is life-threatening and leads to death in many cases. In multiply injured patients, thoracic trauma constitutes the third most common cause of mortality after abdominal injury and head trauma. Furthermore, 40–50% of all trauma-related deaths within the first 48 h after hospital admission result from uncontrolled hemorrhage. Physical trauma and hemorrhage are frequently associated with complex pathophysiological and immunological responses. To develop a greater understanding of the mechanisms of single and/or multiple trauma, reliable and reproducible animal models, fulfilling the ethical 3 R’s criteria (Replacement, Reduction and Refinement), established by Russell and Burch in ‘The Principles of Human Experimental Technique’ (published 1959), are required. These should reflect both the complex pathophysiological and the immunological alterations induced by trauma, with the objective to translate the findings to the human situation, providing new clinical treatment approaches for patients affected by severe trauma. Small animal models are the most frequently used in trauma research. *Rattus norvegicus* was the first mammalian species domesticated for scientific research, dating back to 1830. To date, there exist numerous well-established procedures to mimic different forms of injury patterns in rats, animals that are uncomplicated in handling and housing. Nevertheless, there are some physiological and genetic differences between humans and rats, which should be carefully considered when rats are chosen as a model organism. The aim of this review is to illustrate the advantages as well as the disadvantages of rat models, which should be considered in trauma research when selecting an appropriate in vivo model. Being the most common and important models in trauma research, this review focuses on hemorrhagic shock, blunt chest trauma, bone fracture, skin and soft-tissue trauma, burns, traumatic brain injury and polytrauma.

## Background

Trauma is the leading cause of mortality in humans aged below 40 in high-income countries [1, 2]. Patients involved in severe accidents frequently suffer more than one severe traumatic insult, which is also described multiple trauma or polytrauma. Polytrauma is defined as significant injuries of three or more points on the abbreviated injury scale (AIS) in two or more different anatomic abbreviated injury scale (AIS) regions in conjunction with one or more additional variables from the following five physiologic parameters: age, hypotension, unconsciousness, acidosis and coagulopathy [3]. In multiply injured patients, thoracic trauma constitutes the third most frequent cause of mortality after abdominal injury and head trauma [4]. Furthermore, 50% of all trauma-related deaths within the first 48 h after hospital admission result from an uncontrolled hemorrhage [5]. Physical trauma is frequently associated with a complex immunological response [6] and overwhelming activation of the complement system as well as the associated release of pro- and anti-inflammatory mediators [7]. To model this complex interplay, reliable in vivo systems are required. Basic scientific approaches in trauma research range from zebra fish to nonhuman primates [8, 9].

Small animal models are most frequently used in trauma research, with the aim to improve and develop a basic understanding of the complex posttraumatic regenerative and inflammatory mechanisms. When modeling trauma in rats, adequate analgesia and anesthesia is applied to comply with ethical fundamentals. Furthermore, researchers have to consider that any, even very limited, mental stress or pain could compromise the experimental results and quality of scientific findings and, therefore, have consequently to be eliminated [10]. Post-surgical pain is normally attenuated by opioid analgesics, including buprenorphine and transdermal fentanyl administration [11, 12].

There are multiple rat trauma models, dealing with different injury patterns, including hemorrhagic shock (HS), chest trauma, bone fractures, tissue trauma, burn injury, traumatic brain injury (TBI) or any combination thereof [13–19]. In polytrauma rat models, different single traumata are combined dependent on the objective of the investigation. According to the continuously increasing numbers of rat trauma studies in medline-listed publications, using rats for trauma modeling is well accepted by the scientific community. Rat models have become a powerful tool for research on many aspects of trauma. The most frequently used rat strains, all outbred, are Sprague–Dawley in modeling multiple trauma, TBI, concussion, HS, long bone fracture and blunt chest trauma, followed by Wistar and Long Evans strains.

The search engine of the National Library of Medicine, pubmed, displayed 61213 (September 2018) results for “rat and trauma”. *Rattus norvegicus* was the first mammalian species domesticated for scientific research, dating back 1830 [20]. In 1903, Bateson used rats to demonstrate that rat fur color obeys Mendelian laws [20]. In biomedical research, rats became the most frequently studied animal model [21]. One of the first genetic studies was performed in rats [22]. The first rat inbred strain was established by King in 1909 [23]. Presently, there are more than 200 inbred strains of *R. norvegicus* available [24]. The rat genome was the third complete mammalian genome deciphered. Comparison to the human genome revealed that while the rat genome encodes a similar number of genes, this genome (2.75 gigabases, Gb) is smaller compared to the human genome (2.9 Gb). However, there are some important further differences. Humans have 23 pairs of chromosomes, whereas rats have 21 [25]. All human genes known to be associated with disease have corresponding orthologues in the rat genome, but their rates of synonymous substitution are significantly different in the remaining genes. The overall orthologues genomic regions in rats and humans correspond to 46%, whereas the ‘disease orthologous regions’ correspond to 76% [25]. Regarding immune-system diseases, studies of the respective rodent genes are less relevant compared to other pathophysiology disease systems, because of the rapid diversification of functions of the immune systems of rodents and humans [25]. Even so transgenic modifications in the rat genome allowed an opening of the rat genomic tool box and provided new opportunities to mimic human pathologies and diseases in rats, including hypertension, atherosclerosis, HIV-related pathologies and Huntington’s disease [26].

The Glue Grant Program has improved our understanding about how humans respond to injuries [27]. This program includes data sets of the genomic and proteomic responses to serious, potentially lethal injuries, which were analyzed mainly in the plasma of trauma and burn patients [27]. These findings enable a better modeling of organ failure, predictions for a patient’s outcome and the development of new therapeutics. Nevertheless, there exist anatomic, physiologic and pathophysiologic differences between rats and humans, which have to be considered when planning and interpreting rat trauma models in translational research, particularly when in combining single trauma models [28]. With the aim to increase translational success in trauma research, this review aims to highlight similarities and differences of rats and humans with regard to trauma.

## Coagulation system

The blood coagulation system of the rat has long been investigated. Because this system is extremely rapid in the rat, this special property was used for the application of a specific rodenticide, influencing and acting on the coagulation system by disrupting vitamin K metabolism [29, 30]. Since 1948, the compound ‘warfarin’ has been successfully marketed as a rodenticide. Later, this substance was used as a therapeutic anticoagulant in the clinic and was preferred to other anticoagulants because of its special and beneficial properties. Therefore, the substance was approved as a therapeutic substance for humans in 1954 [29].

In humans, severe hemorrhage following trauma accounts for 40–50% of deaths [5, 31]. During the resuscitative phase, warmed intravenous fluid administration, appropriate transfusion of blood and blood products in combination with surgical control of life-threatening hemorrhage and damage control operations supplemented by angiographic bleeding control is performed according to the Advanced Trauma Life Support (ATLS) guidelines in humans. Trauma-induced coagulopathy (TIC) has been shown to increase with the injury severity score (ISS) [32]. Furthermore, acute traumatic coagulopathy (ATC) dramatically increases the blood loss during trauma. ATC develops very rapidly within the first 60 min after trauma and is also associated with increased patient mortality [33–35]. An animal model of ATC was developed in Sprague–Dawley rats, mimicking the specific clinical scenario [36]. ATC in this rat model was similar to human ATC in terms of temporality, type of injuries, compensation mechanisms and coagulation impairments. Moreover, compensatory mechanisms, maintaining blood pressure and homeostasis were similar in this model in rats and humans [36]. In both rats and humans, the Bezold–Jarisch reflex is activated after ATC, increasing end-diastolic volumes and cardiac output [36, 37]. During ATC, values for the activated partial thromboplastin time (aPTT) and prothrombin time (PT) are comparable between rats and humans [35, 36]. However, quantitative results in blood coagulation are not transferable between rats and humans because of species-specific differences in concentrations of certain clotting factors in the serum [36]. Coagulopathic bleeding needs to be addressed by restoring normal hemostatic physiology. In humans, the PT, aPTT, thrombin time (TT) and fibrinogen concentration are screening parameters to evaluate the main coagulation pathways [38]. By comparing clotting factors and fibrinolytic parameters in human plasma and samples from rats and other animals, fibrinogen, alpha 2 antiplasmin and antithrombin III were in the range of human plasma. By contrast, coagulation factor (F)V, FII, FXII and FXIII in rats were elevated while FVIII, XI, X and XI were reduced compared to pooled human citrated plasma from healthy donors [39]. The platelet count in rats ranges between 500 and 1.300 × 10^9^/L [40, 41] compared to only 150–400 × 10^9^/L in humans [42]. Regarding rotation thromboelastometry (ROTEM) parameters, the clotting time (CT) without thrombin stimulation is three-times longer in humans (595 s) compared to rats (207 s) [43]. Thromboelastometry in a non-activated thromboelastometry (TEM) test also displayed a shorter clot formation time (CFT) and longer maximum clot firmness (MCF) parameters in rats compared to humans [43]. Moreover, platelets were at least four-times less responsive to thrombin compared to humans when aggregometry was used [44]. Furthermore, in an experiment with controlled HS in Sprague–Dawley rats, a prolongation of the plasmatic PT, aPTT and CTs indicated for early and progressive hypocoagulopathy after controlled hemorrhage and shock [45]. Thereby, the early hypocoagulopathy was found to be a two-step process, with platelet dysfunction first followed by fibrinogen impairment, which is also similar in humans [45–47]. Lemini et al. evaluated gender differences in Wistar rats, and coagulation analyses demonstrated differences in the PT, aPTT, TT and fibrin values [48]. Similarly, gender differences in the coagulation system have been shown in humans [49]. Furthermore, posttraumatic analgesia, including buprenorphine, has been shown to interact with the coagulation system: buprenorphine analgesia has been shown to be associated with coagulopathy and increased plasma fibrinogen in healthy rats [12]. Moreover, when modelling blood coagulation in rats, differences in the coagulation system of different rat strains should be carefully considered. Rats of the Fisher and Wistar strains were demonstrated to develop severe hemorrhage in various organs after being fed a vitamin K-deficient diet, in contrast to Sprague–Dawley rats, which did not develop severe hemorrhaging. The animals with severe hemorrhaging displayed abnormal symptoms, including weakness, bloody urine and paralysis [50].

Therefore, because of the extensive species-specific differences between humans and rats, the research on blood coagulation is limited. Although there are some differences, including concentrations of coagulation factors in the serum, platelet count and platelet responsiveness to thrombin, the rat represents a good model for studying trauma-induced coagulopathy because of the highly relevant comparability to the human coagulation system. However, some species-specific limitations in the coagulation system of rats, including the CT, CFT and MCF, need to be considered for a high level of clinical transferability of the model. Summary of modeling the coagulation system in rats is provided in Box 1.

**Box 1:**
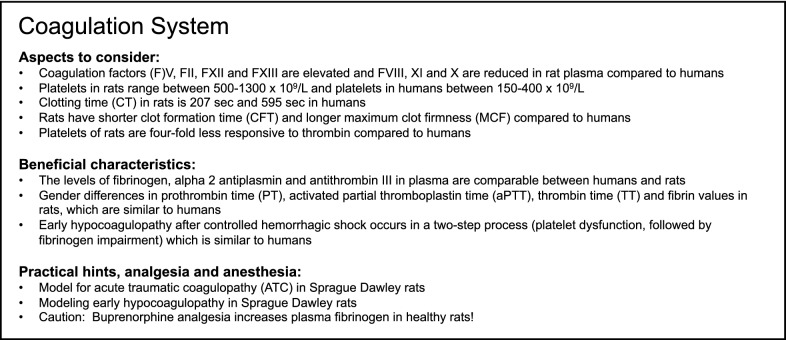
Summary of aspects to consider and beneficial characteristics as well as of practical suggestions, analgesia and anesthesia for modeling the coagulation system in rats

## Hemorrhagic shock (HS)

A total of 3161 (09/2018) pubmed articles were found while searching “rat hemorrhagic shock”. Of these studies, 80% used male rats, 2% females and 18% of unknown gender. Regarding the technique used to induce and maintain HS the majority of the studies were performed by tail-tip amputation. Furthermore, uncontrolled hemorrhage was investigated in different rat models: tail-cut and liver punch biopsy correlated best with class I shock, whereas liver laceration and spleen transection models correlated best with class II shock. Although the anesthesia and analgesia applied in all models had an impact, the heart rate declined in all models throughout the experiment [13]. HS was kept pressure controlled, volume controlled or uncontrolled [51]. For resuscitation, there exist several different protocols, including re-transfusion of shed blood, supplementary infusion of Ringers lactate, saline, hydroxyethyl starch (HES) or any combination thereof [52]. For blood withdrawal and monitoring of the arterial blood pressure HR, arterial catheters can be readily implanted. Here, femoral vessels, the carotid artery or jugular vein are frequently used [53]. In contrast to humans [54], during the HS state in rats, the HR did not increase [55, 56]. Therefore, Choi et al. used a coefficient of the lactate concentration and peripheral perfusion to estimate the shock state in rats [55]. Reynolds et al. described a novel comparative hemorrhagic model of shock vulnerability, which was quantified by so-called ‘vulnerability curves’. In this model, HS was induced incrementally and the physiological response to hemorrhage was determined by measuring the relative changes from the baseline of the cumulative blood volume, mean arterial pressure (MAP) and oxygen delivery (DO_2_) during constant-rate hemorrhage, continued to cardiovascular collapse. Here, the lactate level is mostly independent of the rat strain (Wistar-Kyoto, Sprague–Dawley) [57]. However, Reisz et al. measured systemic levels of lactate and succinate in different species and found significantly higher levels in rats than in non-human primates and humans after HS [58]. Furthermore, there are reports of inter-strain variability in rats, which have to be considered in relation to HS experiments. The PaCO_2_, PaO_2_, oxygen content, potassium, sodium, base excess and lactate in arterial blood measurements during HS were described to differ significantly among five investigated rat strains (Brown Norway Medical College of Wisconsin, Fawn Hooded Hypertensive, Dahl Salt-Sensitive, Dark Agouti and Lewis rats) [59]. In accordance with this, earlier studies similarly revealed strain-dependent differences in the survival time after HS in rats. Therefore, the selection of the rat strain for HS studies must be considered [60, 61].

Troy et al. investigated the role of cardiac vagal and cardiac spinal signals in triggering bradycardia and decompensation during HS in rats and found that cardiac spinal signals play an important role in the triggering and progression of the decompensatory response to hemorrhage [62]. In addition to myocardial autonomic dysfunction during HS, increased cardiac endothelial nitrogen oxide synthase (NOS) expression in rats was found to regulate the HR after blood loss [63]. Furthermore, inhibitors of H_2_S biosynthesis, including glibenclamide, partially restored the HR during HS in rats [64]. When working with rats as a model of HS, the nutritional status of the animals should be considered. Following HS, the enteral microbiome is described as an important source of bacterial endotoxemia and developing sepsis, which depends presumably on the chow of the animals [65]. Germ-free animals displayed a significantly higher survival rate 72 h after HS compared to conventional animals [66]. Additionally, anesthesia method appears to play an important role in executing a HS model in rats: some study groups decided to perform a thoracic epidural anesthesia in rats, because it increased mucosal perfusion of the intestine. Furthermore, this technique also prevented systemic acidemia and increased leucocyte rolling after hypotension, which should be considered when implementing a realistic trauma model in rats [67]. Following HS, the intestinal perfusion flow was also dependent on the gender of the rats, because the perfusion was significantly decreased in males after HS compared to female rats [68]. In agreement with this, Li et al. [69] described that (pre-menopausal) women and female rats displayed a lesser decrease in vascular responsiveness after traumatic shock than older men and rats of the same age, respectively. In pigs, the reactivity of renal vessels was impaired after HS, whereas in rats it increased slightly in the early phase of shock and subsequently decreased gradually [70, 71]. Summarizing, the critical point of HS studies is frequently the microvascular response, which varies across animal species, between the sexes and observed organs and because of factors like anesthesia. Taken together, for studying trauma-induced HS, rat models offer many advantages. Because of the high level of rat model standardization and their large blood volume, the blood pressure and volume can be monitored during hemorrhage, leading to reliable results and reduced standard deviations. Additionally, their large blood volume allows a continuous hemodynamic monitoring as well as repetitive and detailed blood analysis. Moreover, it is technically relatively easy in rats to insert catheters to monitor the level of hemorrhage because of their large and anatomically readily accessible vessels. However, an appropriate HS model in rats should be chosen with deliberation and must be adapted to the aim of the study, particularly because of the manifold variables that influence the microvascular response after blood loss. Summary of modeling hemorrhagic shock in rats is provided in Box 2.

**Box 2:**
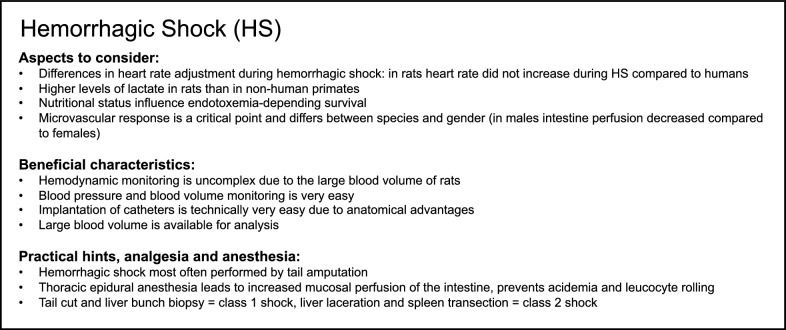
Summary of aspects to consider and beneficial characteristics as well as of practical suggestions, analgesia and anesthesia for modeling hemorrhagic shock (HS) in rats

## Blunt chest trauma

Approximately 25–50% of all injuries involve thoracic trauma [72]. The most frequently used small animal models for blunt chest trauma are captive bolt handgun [14], weight drop (maximum energy equivalent 2.45 J, mortality 33%) [73] and blast wave [74–76], resulting in pulmonary contusion and in systemic and local inflammation. To prevent an associated cardiac injury, in weight-drop models an additional protective shield is utilized [73, 77], whereas cardiac injuries can be detected in models induced by blast wave [78]. Functionally, correlation between the volume of the lung contusion and dysfunction are similarly limited in rats and humans [79, 80]. Clemedson and Pettersson already described in 1953 the mechanical forces that are relevant for lung contusion, with subsequently disrupted alveoli and small airways by shearing forces as well as for stripping of alveolar tissue from heavier hilar structures, caused by acceleration at different rates [81]. Increased cytokine and chemokine concentrations in bronchoalveolar lavage fluid (BAL) have been found after trauma in rats as well as in the human situation [73, 82, 83]. Neutrophil infiltration was detected in lung tissue in rats after blast injury [74], whereas neutrophil depletion significantly reduced lung injury based on BAL albumin concentrations post contusion [73]. Furthermore, chemotactic and phagocytic activity of alveolar macrophages was increased after blunt chest trauma, which is again similar to humans [82, 84]. Modeling blunt chest trauma in rats is well defined and standardized, however, there are some differences between rats and humans to be considered when interpreting data. One divergence is based on differences in toll-like receptors (TLRs) and thus in the recognition of endotoxins and damage-associated molecular patterns (DAMPs) [85]. In the extracellular domain of TLR4, humans and rats share only 61% total amino acid similarity [86]. Moderate levels of TLR4 expression were detected in human lungs, whereas human alveolar epithelial type II cells and alveolar macrophages have been shown to mainly express TLR2 [87]. In contrast to human dendritic cells (DC), all rat DC subsets and monocytes from Sprague–Dawley, Lewis and Brown Norway rats express TLR4 [85]. Therefore, when using trauma rat models for the prediction of human responses to TLRs agonists, scientists should be aware of the inherent limitations of rat studies. Even the discussed presence of pulmonary intravascular macrophages (PIM) [88], constitutive or inducible in humans and in rats respectively, may contribute to species-dependent differences in the sensitivity to endotoxin-induced lung injury [89]. Additionally, previous data have shown similarities in the histological appearance of CD68-positive intravascular cells in human and rat lungs of hepatopulmonary syndrome [90]. Lipofibroblasts are another cell population known in the lungs of rats (and mice), but is still under debate for human lungs [91]. Furthermore, nitric oxide (NO) is an important mediator of numerous physiologic and inflammatory processes in the lung. Constitutive NOS (cNOS) has been found in human lung nerves and large-vessel endothelium, but was lacking in the airway and alveolar epithelia. In rats, cNOS was found in lung nerves, endothelium and alveolar epithelium [92]. Inducible NOS (iNOS) was expressed in human alveolar macrophages during chronic inflammation and, quite similarly, in rat macrophages after lipopolysaccharide (LPS) treatment [92]. Rat and human neutrophils have been shown to produce comparable amounts of NO, but much less than rodent macrophages [93]. With regard to anatomical differences between rat and human lungs, airway branching in humans is more dichotomous and symmetric, whereas rat lungs are more monopodial [94]. The latter might need to be considered when studying air flow distribution, gas uptake and aspiration. Furthermore, severe blunt chest trauma is also associated with cardiac inflammation and structural alteration of cardiac tissue in rats [78]. Rats with blast wave-induced blunt chest trauma displayed acute cardiac tissue damage as well as increased concentrations of circulating heart fatty acid binding protein (H-FABP). Furthermore, rats exhibited increased local cardiac inflammation by increased interleukin (IL)-1β levels as well as disturbed cardiac gap-junction architecture [78]. Moreover, increased blood levels of the N-terminal pro-B-type natriuretic peptide after blunt chest trauma in rats was correlated with a blunt-induced cardiac trauma [95]. However, when modeling cardiac damage after blunt chest trauma, differences between the respective rat strains as well as gender differences within the same rat strain should be carefully considered. Male Sprague–Dawley, Wistar and Wistar-Kyoto rats have low initial serum cardiac troponin I levels, whereas Spontaneous Hypertensive and Fisher rats have high baseline troponin I levels. Furthermore, the baseline troponin I levels differ within the same rat strain between the male and female. Thereby, female rats of the Spontaneous Hypertensive, Sprague–Dawley and Wistar displayed significantly lower troponin I baseline levels compared to the respective male rats. Moreover, testosterone and estrogen levels might also influence the presence of systemic cardiac troponin I. In male Spontaneous Hypertensive rats, the serum troponin I levels significantly increased after castration, whereas in ovariectomized female Spontaneous Hypertensive rats, the systemic troponin I concentrations were significantly reduced [96].

In summary, the rat blunt chest trauma model has a very high translational potential and shares many similarities with human blunt chest trauma, particularly regarding immunological processes and cardiac events. A summary of modeling blunt chest trauma in rats is provided in Box 3.

**Box 3:**
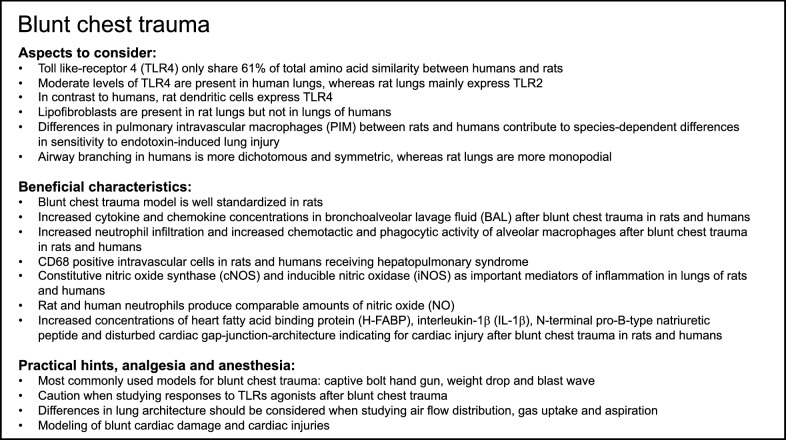
Summary of aspects to consider and beneficial characteristics as well as of practical suggestions, analgesia and anesthesia for modeling blunt chest trauma in rats

## Bone fracture

Rats have been used as a model for bone fracture healing since the 1940′s [15]. Most frequently, femur or tibia fracture is induced in rats. One difference between femur and tibia fracture in rats is the amount of tissue damage, because the femur is completely surrounded by muscles, whereas the tibia at the medial region is only covered by skin [97]. An advantage of the rat model in fracture healing research is the availability of many well-established, standardized fracture procedures. According to ethical considerations, fracture healing studies at present are mostly performed on stabilized fractures. Bonnares and Einhorn in 1994 first described intramedullary nailing of the femur in small animals [97], which was transferred to tibia bone [98]. Currently, there are many different fixation methods available in rats similar to devices applied in humans: unlocked intramedullary pin, locking nail, intramedullary compression screw, interlocking nails and locking plates as well as external fixators [99]. Fracture models include osteotomy by an open surgical approach and closed models using three-point-bending fracture devices. Another advantage of the rat model is the increasing number of naturally occurring or artificially manipulated strains [100] with genetic variations relevant to bone tissue. Because the skeletal phenotype of different rat strains may significantly influence bone healing, this needs to be particularly considered in models like the spontaneous dwarf rat with growth hormone (GH) deficiency [101] and the Komeda miniature rat Ishikawa caused by mutation in Prkg2 [102]. Furthermore, many disease models are available in rats closely mimicking the clinical situation of delayed fracture healing in humans, for example, ovariectomy of rats to induce post-menopausal osteoporosis and subsequent delayed fracture healing [103, 104].

One disadvantage of the rat model is that its bone structure is more primitive than in humans because of a lack of Haversian systems [99]. However, bone healing in rats via resorption cavities has been shown to be similar to Haversian remodeling in larger animals and humans [105]. Single studies report inter-strain differences in hip fragility of rats, which could influence biomechanical outcome after fracture. Comparison of the femoral bone structure of Copenhagen 2331 rats showed considerable differences in femoral neck structure, bone density and mineral content compared to Dark Agouti rats, despite similar body mass and biomechanical properties at the femoral midshaft and lumbar spine [106]. Such inter-strain variabilities of bone structure need further attention in trauma research.

Furthermore, gender [107] and age [108] are important variables for bone research. In rats, bone growth continues much longer after sexual maturity than in humans [109]. In younger rats, fracture consolidation is much more rapid compared to older animals, which corresponds to age-dependent bone healing in humans [110, 111]. When comparing male and female rats of the same age, male animals have a greater body weight, resulting in greater interfragmentary movements at the fracture site. In rats, the bone-healing process is completed within 5 to 6 weeks [112] and is highly dependent on biomechanical conditions of fracture stabilization, as was also shown in humans [113].

Taken together, when studying bone fracture healing in rats, there are several surgical techniques available. An increasing number of genetic manipulations in rats affecting skeletal phenotype allows more specific investigations. However, rat strain, age and gender should be considered critically. Overall, rat disease models, like ovariectomy and enhanced age, clearly demonstrated that the rat is able to mimic pathological processes in patients to a close extent, and is, therefore, a valuable tool to gain a deeper understanding of the complex bone-healing process [103, 104], although bone structure differs from humans. A summary of modeling bone fracture in rats is provided in Box 4.

**Box 4:**
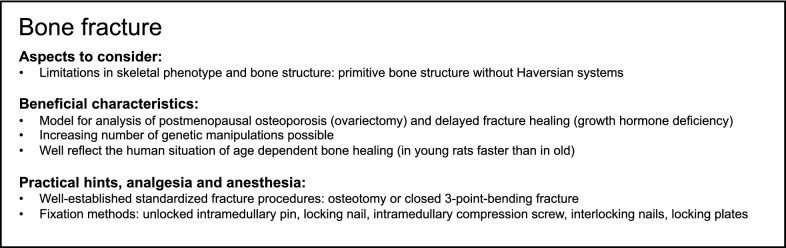
Summary of aspects to consider and beneficial characteristics as well as of practical suggestions, analgesia and anesthesia for modeling bone fracture in rats

## Skin and soft-tissue trauma

Trauma is frequently associated with damage of skin and soft tissue. There is an enormous difference between the humans and rats, particularly in skin appearance [114]. Rats’ skin clearly has little similarity with humans. The rat was also described as a “loose skinned animal” [16], because of the very limited adherence strength to the structures below and the skin’s elasticity [114]. Additionally, rats are able to convert l-gluconogammalactone to vitamin C. Vitamin C is very important for the synthesis of collagen and, therefore, also for wound and soft-tissue healing. Furthermore, the epidermis and the dermis of rats are thinner than in humans, which could be challenging for suturing. Interestingly, rats are frequently used as a special model for a part of the complex system of wound healing in humans: wound contraction. “Wound contraction is considered to be the primary healing method of rats as opposed to re-epithelialization seen in humans” [16]. The panniculus carnosus muscle is responsible for wound contraction and collagen formation in rats [114, 115]. Furthermore, the risk of wound infection is lower in rats compared to humans because of the reduced healing time [16]. In skin and soft-tissue trauma, differences in rat strains were observed between Brown Norway, Lewis and Wistar rats. Thereby, the different rat strains displayed variations in the severity of skin lesion after exposure to hexachlorobenzene, which was associated with activation of the immune system in the respective rat strain [116].

Taken together, rats could be used as trauma models for very specific questions regarding skin and tissue traumas. However, several limitations, as mentioned above, should be considered when rats are used for wound-healing studies to ensure valid and reliable results. A summary of modeling skin- and soft tissue trauma in rats is provided in Box 5.

**Box 5:**
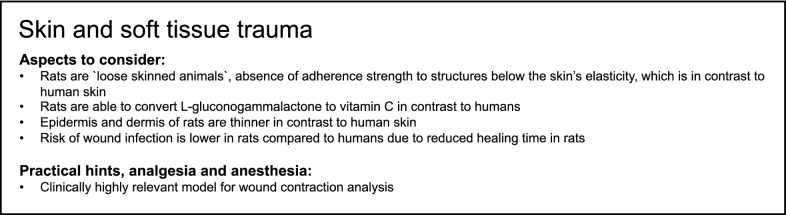
Summary of aspects to consider and beneficial characteristics as well as of practical suggestions, analgesia and anesthesia for modeling skin and soft-tissue trauma in rats

## Burn injury

Burn injury is one of the most weakening traumas affecting humans. According to the World Health organization (WHO), more than 300,000 deaths globally are caused annually by burns [117]. For investigation of burn injuries in rats, various burn injury rat models are available. The most frequently used burn model in rats is the scalding burn model. Here it is possible to determine the exact exposed surface area of the skin [16]. The temperature and the immersion time are variable and vary from study to study. The major drawback of this model is that the rat in contrast to human is able to cope with hypermetabolism. Hyperglycemia frequently occurs during the early post-burn phase in humans. Therefore, it could be very complex to induce additional infections in rats, mimicking post-burn sepsis, which frequently occurs in humans [118]. Furthermore, strain differences should be carefully considered when trying to perform lipopolysaccharide (LPS)-induced sepsis in rats. Brown Norway and Lewis rat strains showed different responsiveness to LPS, which was modulated by the liver of the animals [119]. In another full-thickness skin burn model, the temperature and the burned area are variable. The major drawback of this method is the lack of a homogenous uniform burn depth [16]. In another rat burn model, radiant heat was used [120]. In this model, the heating source does not make direct contact with the rat skin and the heat dispersion is very constant. When conducting burn- or wound-healing studies in rats, it should always be critically kept in mind that differences in human and rat skin are also present internally [16, 17]. This is described in the previous chapter. In conclusion, rat models for studying burn injuries allow specific investigations of local burn injuries, which are of high clinical relevance for traumatic issues. A summary of modeling burn injury in rats is provided in Box 6.

**Box 6:**
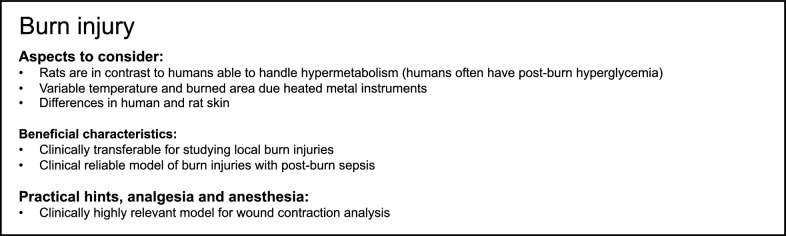
Summary of aspects to consider and beneficial characteristics as well as of practical suggestions, analgesia and anesthesia for modeling burn injury in rats

## Traumatic brain injury (TBI)

Severe injury to the central nervous system (CNS) accounts for a third of all trauma-related deaths followed by CNS injury with additional exsanguination (17%) [5]. Mild TBI (concussion) occurs most frequently after trauma [121]. The most frequently used methods to initiate TBI in rodents are the lateral fluid percussion injury (LFP), controlled cortical impact (CCI) and weight-drop impact. LFP results in reproducible pathological changes similar to those observed in human head injury [18, 122]. Histopathological findings were subarachnoid and intraparenchymal hemorrhage [122]. Furthermore, CCI reflects a severe form of TBI, with skull deformation and related cortical compression. In this model, mechanical force, velocity and depth of skull deformation can be controlled, and thus it provides important advantages for standardizing research on TBI. The pathological findings are diffuse axonal injury, subdural hematoma, brain edema, elevated intracerebral pressure, reduced perfusion, metabolic changes, blood–brain dysfunction and coma [53].

A weight-drop impact induces both apoptotic and necrotic neuronal cell death by activating proinflammatory mediators, caspases and members of the Bcl-family [123]. A metal cylinder falls from a fixed height (approx. 2 cm) onto the dura. The gravitational force induces contusion, cortical cell loss, edema, blood-barrier dysfunction and apoptosis. Histologically, bilateral damage of neurons, axons, dendrites and microvasculature is observable [53, 124].

Another rat model of TBI is the cryogenic injury model, leading to a focal brain lesion [125]. In this model, the brain injury is induced by applying a cold rod to the exposed dura [126]. Different injury severity can be achieved by varying the exposure time of the cortex to the rod [127]. Standardized lesions caused in this model are clearly confined and highly re-producible [125]. However, this model lacks the formation of diffuse axonal injuries, which frequently occur in human brain injuries [128]. Another rat brain injury model, which became increasingly important during recent years, is blast-induced brain injury. Thereby, rats were mainly exposed to blast waves of different intensities, mimicking blast waves caused by explosions [129]. When modeling TBI in rats, differences in rat strains should be carefully considered. The response to injury strongly varies between different rat strains, which might contribute to differences in viability among studies. Sprague–Dawley and Fisher rats have received lateral fluid percussion injury. Thereby, Fisher rats exhibited a greater mortality rate and longer duration for regeneration of the righting reflex. Moreover, differences in motor and cognitive abilities after trauma were shown in the two different strains. Thereby, Fisher rats displayed greater motor deficits but performed better in cognitive tests following injury compared to Sprague–Dawley rats. However, Fisher rats showed increased caspase-3 expression, higher intracranial pressure and prolonged seizure activity compared to Sprague–Dawley rats [130].

When modeling TBI, scientists frequently analyze behavioral reactions [131]. However, there are differences between rodents and humans concerning physiological and behavioral responses to TBI [132]. The rodent brain is inappropriate for representing the more complex human cortex. Although there are many differences in brain anatomy and complexity, there are also some analogies, for example, in cerebrovascular parameters [123]. Cordeiro et al. highlighted that the human brain ratio of white:gray matter differs from rats; because the amount of white matter is significantly smaller [133]. Codeiro et al. additionally indicated that human intracerebral hemorrhage is neither an insult at a single spot in the brain nor restricted to one anatomical region. However, the induced traumata only inflicted one region in the brain, which differs from a real traumatic injury [133]. Rats’ brain edema reaches the full extension within a few days of brain injury, whereas in humans, brain edema persists for weeks [133]. There are many well-established neurological tests, including the Morris water maze and the Open-Field-Test, which are applicable to rats based on their capability to learn quickly. Following concussion, the activity of exploration is significantly reduced [134]. Furthermore, Eakin et al. described mice as swimming more poorly than rats, giving rats a clear advantage in different research settings. Sham animals of this study performed significantly better in neurological tests than animals with a weight-dropped-induced TBI [135]. The play fight behavior was significantly reduced in animals with concussion. The male rats with TBI decreased their play fight regardless of their mates’ conditions. By contrast, female rats initiate less social interaction with mates with TBI. This ability to interpret emotions and behavior is located in the frontal lobes of the brain, which is a common target of concussion in humans. The elevated female sensitivity might lead to a greater risk for depression, anxiety and loneliness after TBI [136]. The Open-Field-Test is particularly designed for rats and observes the spontaneous intension of exploration. The animal is placed in a box, movements are monitored by a video camera and the overall distance travelled in a defined time period is automatically quantified. Following concussion, the activity of exploration is significantly reduced [134].

Traumatic brain injury is associated with a neuroinflammatory response involving brain-resident immune cells, including microglia and reactive astrocytes present at the impact site [137, 138]. In this cellular response, similarities between human and rat TBI were described. For example, microglia activation, involved in the clearing of myelin debris, occurs both in rats and humans [139–142]. A difference between human and rat TBI might exist with regard to the duration of the neuroinflammatory response. In the rat, while neuroinflammation occurs early after TBI, this response is rather transient, whereas after human TBI, microglial activation can persist for several years [141, 142]. Microglia and other immune cells in the TBI impact site are known to secret interleukins, chemokines and other factors [137, 138]. In this cellular response, similar expression of selected molecules, including interleukins (IL-18) and tissue inhibitor of metalloproteinase 1 (TIMP-1), were reported in both human and rat TBI [143–145]. Therefore, although differences in TBI-associated cytokine and chemokine profiles might exist between humans and rodents, some molecular patterns are conserved.

An important aspect in TBI research is the identification of prognostic biomarkers from, for example, blood and cerebrospinal fluid samples. Interestingly, in human and rat TBI fluid samples, a similar biomarker profile was observed [146, 147]. This included proteins like Tau, S100B and glial fibrillary acidic protein (GFAP). Given these similarities, rat TBI models offer the opportunity to identify cellular sources and time courses of biomarker secretion and allow for the correlation of biomarker expression with functional recovery progression after TBI.

TBI is associated with several comorbidities in the years that follow the initial TBI event. These include enhanced frequency of neurodegenerative diseases, for example, Alzheimer’s disease, but also epileptic seizures [148]. Here, animal models, including rat TBI models, allow for experimental strategies to determine the cellular and molecular interactions between the initial TBI event and, example, subsequent seizures, and thus enable the development of potential therapeutic interventions [149]. The rat is well suited to analyze these interactions, and seizure occurrence is reported in several TBI models [149, 150].

An important molecular hallmark initiated by TBI-injured neurons is the induction of gene expression. In particular, rapid (within minutes) induction of so-called immediate early genes (IEGs), like c-Fos and c-Jun, has been reported in several rodent TBI models, including rats [151–153]. Because many IEGs encode transcription factors, a subsequent delayed gene expression program is initiated, which might contribute to neuronal protection after TBI. Notably, similar to rats, induction of selected IEGs, including c-Fos and c-Jun, was also reported in postmortem human TBI brain tissue [154–156]. Therefore, from a molecular perspective, gene expression responses in TBI-injured human and rat neurons appear to some extent to be conserved. Such findings, suggesting conserved TBI-associated molecular responses across different species underline the suitability of rodent models to investigate cellular and molecular TBI responses and to test pharmacological options to accelerate post-TBI recovery. A summary of modeling TBI in rats is provided in Box 7.

**Box 7:**
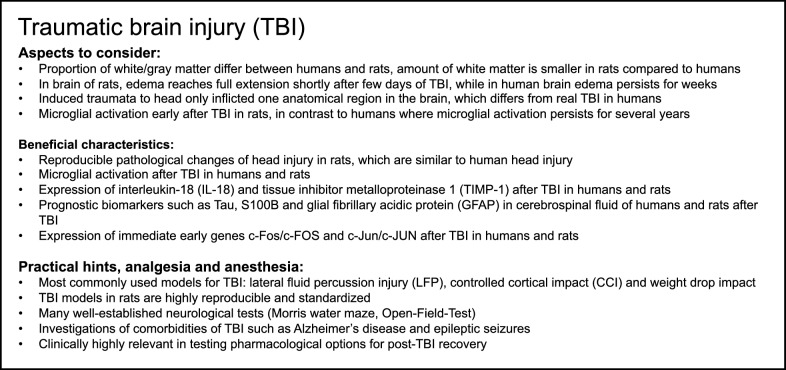
Summary of aspects to consider and beneficial characteristics as well as of practical suggestions, analgesia and anesthesia for modeling traumatic brain injury (TBI) in rats

## Polytrauma

Polytrauma is defined as a combination of multiple severe and simultaneous injuries to more than one body region or organ system [3, 157]. The effects of the combined injuries on the patient are not comparable with a single trauma, and the mortality of patients affected by polytrauma increases significantly [28, 158]. Although certain singular injuries, including severe TBI and hemorrhage, are clearly associated with higher mortality rates, the combination of multiple injuries aggravates further the outcome [159]. This is caused by the complex posttraumatic immune response, which is a key driver of late post-injury complications and fatal outcome rates after trauma [160, 161]. Interestingly, despite improved treatment strategies with regard to traumatic injuries as well as to the posttraumatic immune response, both mortality and disability rates still remain alarmingly high [162]. Therefore, a reliable in vivo model is necessary to investigate the physiological and pathophysiological responses to polytrauma. The most frequently applied in vivo models to addressing polytrauma do not actually represent polytrauma, because they mainly involve two different insults and thus depict double-hit trauma models. Polytrauma in rats can include any combination of burn injury, fracture, hemorrhage, trauma to the extremities and soft-tissue trauma among many others. In a recently established polytrauma rat model, chest trauma, closed head injury, tibiae/femur fracture and soft tissue trauma were combined [28]. In these polytraumatized rats, a significant systemic and intrapulmonary release of cytokines, chemokines and complement anaphylatoxins compared to rats with isolated injuries or selected combinations of injuries have been observed. Therefore, the authors provided evidence that a double-hit trauma model is of limited suitability to represent a clinical polytrauma in patients. Denk et al. included HS to the above described polytrauma model to further increase its clinical relevance, however, the experiments were performed on mice [163, 164]. The authors described the detection of specific barrier molecules in a murine polytrauma model and in patients after polytrauma, which appeared to be injury-pattern and time dependent. Therefore, this model is useful to assess posttraumatic barrier dysfunction. However, to date, a long-term polytrauma model in rats, which may be applicable, for example, to study complications like organ failure and sepsis, has not been established. Other issues, including the timing of the application of various traumatic injuries, should also be considered. In an interesting double-hit model, burn injury with soft-tissue and gastrointestinal-tract trauma were combined [19]. Unlike many double-hit models, the authors applied the injuries simultaneously in their model, an approach that actually mimics the setting of the polytraumatized patient. They also confirmed that there were differences between the single injuries and polytrauma in the maintenance of blood glucose, body temperature, body weight, hepatic mRNA and circulating levels of tumor necrosis factor, IL-1β and IL-6 and hepatic endoplasmatic reticulum stress [19]. Therefore, the authors confirmed that models utilizing combinatorial injuries are needed to more accurately model the human condition. Another polytrauma model includes animals that were subjected to a laparotomy plus burn and single puncture of the cecum injuries [165]. The authors concluded from their data that their model of polytrauma is straightforward to perform and highly reproducible, making it a useful model for studying the multifaceted early pathophysiology following polytrauma. Furthermore, they provided evidence that using a double-hit model to represent polytrauma is of limited validity, because the findings again suggest a complex pathophysiological response to polytrauma, and indicate that the mechanisms leading to the development of insulin resistance vary depending upon the type of injury [165]. Interestingly, their data are unlike those found in more severe single-injury models or those observed following the combination of trauma and hemorrhage. Darlington et al. developed a rat model of polytrauma and hemorrhage that is coagulopathic and displays a prolongation of the PT and platelet dysfunction that closely parallels clinical findings in human trauma patients [166]. In their model, polytrauma was induced by damaging the small intestines, the right and medial liver lobes and the right leg skeletal muscle and inducing fracture of the right femur and hemorrhage. Therefore, it remains indisputable that the choice of the appropriate model has to clearly address the scientific question being asked. Additionally, data from some double-hit models, which are termed as “polytrauma”—because they include more than a single injury type, have to be very carefully interpreted when referring to clinical polytrauma.

By contrast the most-described experimental in vivo models in rats barely meet the definition of polytrauma according to an ISS ≥ 16. Here again, when referring to the human situation, the in vivo model should include three or more traumatic injury patterns, involve life-threatening injuries, including brain, chest or abdomen injury, and exert an ISS > 15. While Weckbach et al. and others, as described above, have compared their experimental polytrauma model with different double-hit models, and on the one hand found that the injury pattern matters, while others on the other hand underlined the undeniable relevance of, for example, HS after experimental polytrauma, as demonstrated by Denk et al. in a mouse model. However, there is another important difference compared to the human situation after trauma, which should also be considered when interpreting the data. Contrary to the human situation, experimental polytrauma is performed under controlled conditions without awakening the animals until sampling.

Taken together, many double-hit models but only a few models of polytrauma combining different injury patterns are applied to study and understand the basic pathophysiology of polytrauma. Modeling the complex injury patterns of polytrauma and the subsequent immune response remains both difficult and challenging, because no single animal model is able to fully represent the diversity of polytrauma as observed in the human situation. Importantly, experimental polytrauma models should imply as little injuries as possible to reduce the harm of animals according to the criteria of the Replacement, Reduction and Refinement (3Rs). A summary of modeling polytrauma in rats is provided in Box 8.

**Box 8:**
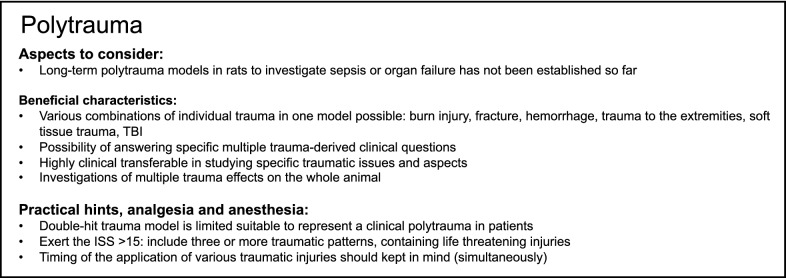
Summary of aspects to consider and beneficial characteristics as well as of practical suggestions, analgesia and anesthesia for modeling polytrauma in rats

## MSC-based cell therapies after trauma

The rat models which are described in this review are also suitable to study therapeutic intervention in trauma. As an example, we describe the emerging field of cell therapies, in particular the intervention with mesenchymal stem/stroma cells (MSC) or MSC-derived effectors.

MSC have been widely used in pre-clinical and clinical research studies on immunomodulation as well as protection or regeneration of damaged tissues (e.g. for treatment of graft-versus-host disease, stroke, brain/nerve injury, bone defects, osteoarthritis) [167, 168]. They are promising candidates for regenerating damaged tissue and modulating deregulated immune reactions. MSC from various tissues (bone marrow, adipose tissue, cord blood and others) and various donor types (autologous, allogeneic, xenogeneic) have been used in these studies. Beneficial effects of MSC were observed in many disease models in rats, independently of tissue source or donor type [169, 170]. The effects of MSC cannot be explained by a single mechanism of action but seem to be mediated by variety of properties of MSC: They can migrate and integrate to the site of tissue injury and respond to DAMPs. MSC can release factors which stimulate the growth and differentiation of neighbouring cells, induce angiogenesis, regulate the development of fibroblasts and endothelial cells, inhibit fibrosis and create an anti-inflammatory, pro-regenerative microenvironment. Paracrine actions include secretion of cytokines, chemokines and extracellular matrix, and release of extracellular vesicles (EV). They can release EVs of various size ranging from exosomes to microvesicles [171]. These EVs contain proteins, organelles, miRNA and mRNA [172, 173]. They constitute an important inter-cellular communication system. It has been demonstrated that EVs in some models exert similar effects than MSC themselves [171].

Several in vivo studies, including rat models, demonstrated the unique immune privilege of MSC which can facilitate their use in an allogeneic and even xenogeneic setting [174–178]. However, the impact of donor type of MSC on the effects is discussed controversial and might differ depending on other factors. In a rat model it has been demonstrated that different routes of administration and different microenvironments can lead to divergent immunogenicity of allogeneic MSC [179].

The systemic administration of human (h) bone-marrow MSC in a rat model of blunt chest trauma reduced the lung injury score 24 h after trauma by at least 50% compared with traumatized rats without MSCs and the MSC treated rats exhibited a lower level of pro-inflammatory cytokines (interleukin [IL]-1B, IL-6) and chemokines (C-X-C motif chemokine ligand 1 [CXCL1], C-C motif chemokine ligand 2 [CCL2]), but a higher tumor necrosis factor alpha induced protein 6 (TNFAIP6) level in the BAL [178]. In this model, hMSCs could not be detected 24 h after injection into traumatized rats in the peripheral blood and no human Alu sequences were detectable in the blood or in lung tissue [178]. Also other publications on rodent models of acute lung injury suggest that MSC effects are mediated via secretion of TNFAIP6 [18], IL-1RN [180] or KGF [181] rather than engraftment and differentiation and long-term persistence of these MSCs. Besides the secretion of soluble factors also the release of EVs can contribute to the beneficial effect of xenogeneic MSCs [182, 183]. Effects of EVs have been demonstrated also in other injury models in rats, e.g. TBI [184], nerve injury [185, 186], pre-term brain injury [177, 182] or burn injury [187].

Many studies confirmed the potential of MSC for bone repair, including a series of rat models (e.g. critical size defects, non-union fractures). Bone repair by MSC has been demonstrated after different routes of administration (locally, mostly with MSC bound to a scaffold [188–192] or systemic injection [193]), different types of MSC (bone marrow or adipose-derived MSCs), ex vivo expanded MSC with/without gene modification (e.g. overexpression of BMP2 [194] or specific micro-RNAs [195, 196]) and with or without specific pre-differentiation towards the osteoblastic lineage [197, 198] and also with MSC-derived supernatant [199, 200] or exosomes [201–204].

A meta-analysis of efficacy of MSC in animal models of traumatic brain injury, including different rat strains (Wistar, Sprague–Dawley, Fisher 344), concluded that MSC therapy may improve locomotor recovery after TBI [168]. For neurological motor function, significant differences were observed in terms of MSC donor type and MSC dose [168]. No significant differences were found in terms of route of administration and tissue source [168].

Overall these studies demonstrate that the rat trauma models can also be used to study therapeutic intervention comparing different interventions (intact MSC from different tissues, MSC-derived soluble factors or MSC-derived EVs) administered via different routes. As demonstrated in several rat models an allogeneic or even xenogeneic cell therapy approach is feasible, thus allowing pre-clinical evaluation of human MSC intended to be developed for clinical use.

## Conclusion

Many trauma models are available in rats, and because they may closely correspond to the human response to injury, several differences between humans and rats should be carefully considered when modeling trauma. For example, in rats there are limitations in the coagulation system and some divergent reaction to massive blood loss compared to humans. To reproduce lung injury after blunt chest trauma in rats, the high NO production in rodents and the lack of pulmonary intravascular macrophages should be taken into consideration. Furthermore, there are some limitations in wound healing after soft-tissue trauma and burn injury between rats and humans. Additionally, rats display certain differences in bone structure and a more rapid bone-healing process compared to humans. Certainly, rats are a good model for TBI research, on the one hand because the neurological tests suggest a high capability to learn quickly and because of their curiosity. On the other hand, there is a lack of a perceptive faculty in rats. Furthermore, when considering polytrauma research, the extent of the simultaneously applied single traumata can be investigated more closely with regard to the whole animal.

In summary, when considering both the advantages and disadvantages of using rat models for trauma research, in planning and performing animal experiments the criteria of the Reduction, Replacement, Refinement (3 R’s), which were published by Russell and Burch in 1959 in ‘The Principles of Humane Experimental Technique’, should additionally always be considered [205]. Animal welfare should always be the first priority together with the reduction of animal pain and suffering. When planning animal experiments, first all potential replacement and alternative reliable experimental methods should be excluded. When there is no alternative, many basic trauma and regeneration processes have been shown to be similar to humans, and thus make the rat a suitable model for translational research.

## Data Availability

Not applicable.

## Abbreviations

AIS: abbreviated injury scale
ATC: acute traumatic coagulopathy
ATLS: Advanced Trauma Life Support
APTEM: activation as EXTEM with aprotinin to inhibit fibrinolysis
aPTT: activated partial thromboplastin time
BAL: bronchoalveolar lavage fluid
CCI: controlled cortical impact
cNOS: constitutive nitric oxide synthase
CNS: central nervous system
CSF: cerebrospinal fluid
CT: clotting time
DAMPS: damage-associated molecular patterns
DC: dendritic cells
F: factor
FACS: fluorescence-activated cell sorting
Gb: gigabases
GFAP: glial fibrillary acidic protein
GH: growth hormone
HES: hydroxyethyl starch
HFABP: heart fatty acid binding protein
HR: heart rate
HS: hemorrhagic shock
IEGs: immediate early genes
IL-1β: interleukin-1 beta
IL-6: interleukin-6
IL-18: interleukin-18
ISS: injury severity score
LFP: lateral fluid percussion injury
LPS: lipopolysaccharide
MAP: mean arterial pressure
MCF: maximum clot firmness
NO: nitric oxide
NOS: nitric oxide synthase
iNOS: inducible nitric oxide synthase
PIM: pulmonary intravascular macrophage
PT: prothrombin time
3 R’s: Reduction, Replacement, Refinement
ROTEM: rotation thromboelastometry
TBI: traumatic brain injury
TEM: thromboelastometry
TIC: trauma-induced coagulopathy
TIMP: tissue inhibitor of metalloproteinase
TNF: tumor necrosis factor
TLR: toll-like receptor
TT: thrombin time
WHO: World Health Organization
